# Recent Advances in the Synthesis and Application of Three-Dimensional Graphene-Based Aerogels

**DOI:** 10.3390/molecules27030924

**Published:** 2022-01-29

**Authors:** Jingyun Jing, Xiaodong Qian, Yan Si, Guolin Liu, Congling Shi

**Affiliations:** 1 Beijing Key Laboratory of Metro Fire and Passenger Transportation Safety, China Academy of Safety Science and Technology, Beijing 100012, China; bdqyjjy@163.com (J.J.); wjxyqxd@hotmail.com (X.Q.); liugl@chinasafety.ac.cn (G.L.); 2 Postdoctoral Research Station of Beijing Institute of Technology, Zhongguancun Smart City Co., Ltd. Substation of Zhongguancun Haidian Yuan Postdoctoral Centre, Beijing 100081, China; siyan_26@126.com

**Keywords:** three-dimensional, graphene-based aerogels, synthetic strategy, application, prospect

## Abstract

Three-dimensional graphene-based aerogels (3D GAs), combining the intrinsic properties of graphene and 3D porous structure, have attracted increasing research interest in varied fields with potential application. Some related reviews focusing on applications in photoredox catalysis, biomedicine, energy storage, supercapacitor or other single aspect have provided valuable insights into the current status of Gas. However, systematic reviews concentrating on the diverse applications of 3D GAs are still scarce. Herein, we intend to afford a comprehensive summary to the recent progress in the preparation method (template-free and template-directed method) summarized in Preparation Strategies and the application fields (absorbent, anode material, mechanical device, fire-warning material and catalyst) illustrated in Application of 3D GAs with varied morphologies, structures, and properties. Meanwhile, some unsettled issues, existing challenges, and potential opportunities have also been proposed in Future Perspectives to spur further research interest into synthesizing finer 3D GAs and exploring wider and closer practical applications.

## 1. Introduction

**Figure 1:**
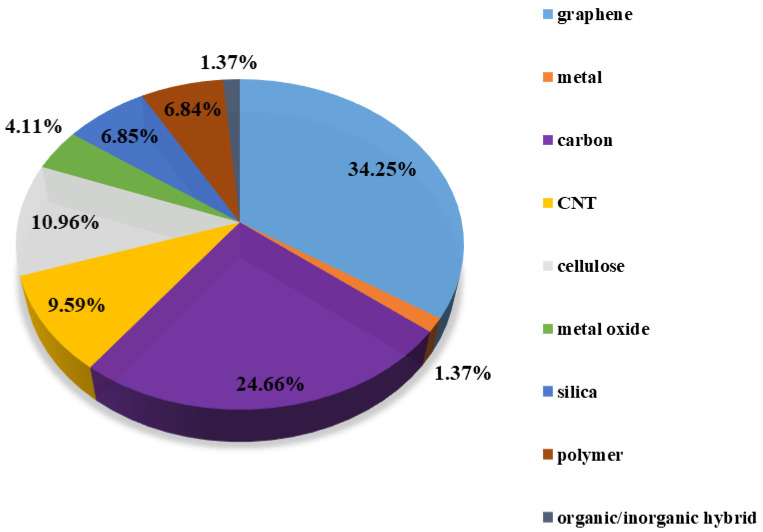
Percentage of aerogels based on chemical component. Data are summarized on the Web of Science from 2010 to May 2020.

Aerogel, generated by the replacement of liquid inside a gel with gas by freeze-drying or supercritical drying technique, was first presented by S. Kistler in the 1930s [1]. As the lightest solid porous material in the world, it has attracted wide attention owing to its three-dimensional (3D) network structure, high specific surface area, extremely low-density, and thermal conductivity [2,3,4]. The precursor of aerogel can be selected among organic polymers [5], inorganic materials [6], and polymeric hybrid materials [7]. At the beginning, it underwent a long slow-development-stage because of the difficulty in synthesis and the lack of application. However, aerogels with richer types and application fields have been flourishing in the past decade. Such a porous material can be an enticing prospect for its application in the fields of aerospace, chemical engineering, construction, electrical equipment, water purification, and biomedicine [8,9,10,11]. Among them, graphene aerogels [12,13], carbon aerogels [14,15], and carbon nanotube (CNT) aerogels [16] are the most investigated topics, comprising more than 60% of the literature studies, as shown in Figure 1.

As the most important component of two-dimensional (2D) carbon-based material, graphene possesses superior thermal conductivity (~5000 W/m·K), high specific surface area (2630 m^2^/g), chemical stability, and high electron mobility, as well as excellent mechanical and optical properties, which has great application potential in many fields [17]. However, their direct application as excellent adsorbents, anode materials, and mechanical devices suffers from limitation, as a serious loss in surface area occurs due to the re-stacking problem resulting from π-π interaction between graphene layers and the Van der Waals force [18].

**Figure 2:**
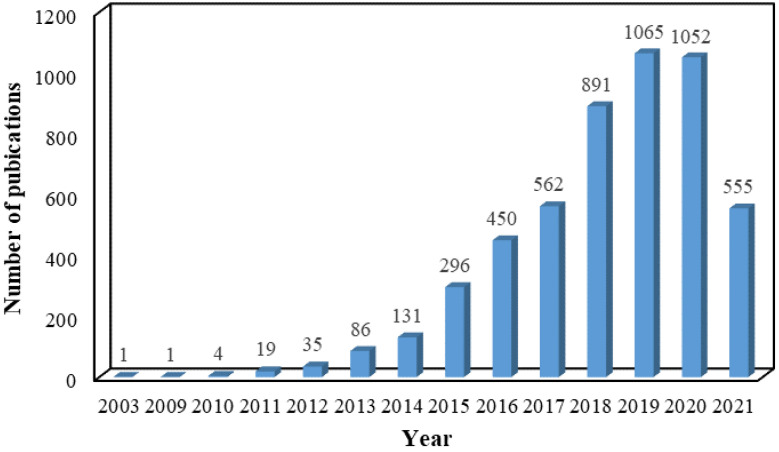
Number of publications about GAs in the past almost two decades. Data are summarized on the Web of Science.

The past several years has witnessed explosive interest in constructing a series of composites based on the versatile platform of graphene. In order to overcome the problem and make full use of the characteristics of graphene sheets, the conversion of 2D sheet to 3D porous aerogel via self-assembling by various methods, including hydrothermal reduction, chemical reduction, crosslinking, and sol-gel processes, is an ideal choice [19]. Due to integration intrinsic properties, including high porosity, conductivity, and feasibility of manufacturing in an industrial scale of graphene and aerogel, 3D GAs have emerged as one of the most exciting and promising materials. The past almost two decades have witnessed a rapid development process in exploring the performance and application of GAs, as reflected in the increasing number of publications collected in the Web of Science database for the search criteria “graphene AND aerogel” (Figure 2).

Although there have been some reviews focusing on synthesis methods, properties, and applications in photoredox catalysis, biomedicine, energy storage, supercapacitor, or other single aspects [17,20] of GA, systematic reviews concentrating on the diverse applications of 3D GAs are relatively scarce. Therefore, it is desired to present a comprehensive review that summarizes the current situation, recent advances, and future prospects in the synthesis and application of 3D GAs to inspire intelligent ideas. This review aims to deliver a comprehensive explanation of the recent advances in the preparation methods and application fields of 3D GAs with varied morphologies, structures, and properties. Meanwhile, some unsettled issues, challenges, and potential opportunities have also been proposed. We hope this review will provide valuable insights into the current status of 3D GAs and be a reliable reference for further research, promoting the practical application of the materials in wide fields.

## 2. Preparation Strategies

With the further development of 3D GAs, expanded preparation approaches such as hydrothermal reduction [21], chemical reduction [22,23], electrochemical synthesis [24,25], self-assembly [26,27], emulsion technique [28], breath-figure method [29], chemical vapor deposition [30], and ink-printing technique [31] have been adopted to fabricate varied and unique microstructures. Among these, hydrothermal or chemical reduction is the most attractive one based on its low-cost and scalable production. Here, we have classified these methods into two major categories: template-free method and template-directed method.

The interfacial interaction and chemical composition of as-prepared functional GO and/or RGO-based aerogel can be characterized by various means. XPS analysis is performed to characterize both the chemical state and atomic ratio of every element in the GO and the graphene aerogels. For the C 1s XPS spectrum of GA, the peaks attributed to carbon atoms connecting with oxygenated groups, such as C–O and O–C=O, have disappeared as the oxygenated species are substantially removed on the reduction of GO to graphene [32]. It is also evidence for the deoxygenation process and successful preparation of GAs. The significant structural changes are also reflected in the Raman spectra. The G band, which resonates at a lower frequency and an increased D/G intensity ratio compared to that of GO, suggests a reduction of the exfoliated GO. The FT-IR spectra gives the information of various functional groups, proving the change from graphene oxide into a graphite-like structure due to the chemical reduction.

The graphene-based aerogels are mainly prepared from a GO precursor via the reduction process. The dispersion of graphene in substrate is always particularly important for improving the performance of aerogel. In order to avoid aggregation induced by strong a van der Waals force, many preparation methods involving covalent and/or non-covalent modification between GO and polymer matrix and appropriate ultrasonic dispersion are employed [33]. The dispersion of graphene can be reflected on the morphology of aerogel characterized by SEM, SEM-EDS and TEM, and the XRD pattern. Furthermore, it is feasible to study the dispersion state of graphene by characterizing the typical properties of aerogels based on the obvious effect of dispersion on performance.

### 2.1. Template-Free Approach 

A template-free method is preferred in practical application due to the relatively simple synthesis procedures, low cost, and easy scaling up [34]. A series of template-free methods have been put forward for synthesizing 3D GA, in which the most typical and dominant is the self-assembly method. Taking advantage of inherent orderly stacking behavior via π-π interaction of graphene and GO nanosheets under appropriate conditions can produce various 3D structures [35,36,37] without the need and limitation of a template, making it novel and appealing. However, it is still important to develop suitable approaches to achieve the assembly and avoid the precipitation of graphene in a parallel arrangement while declining the repulsion forces of GO solution.

#### 2.1.1. Reduction-Induced Self-Assembly

Amphiphilic GO consists of an abundant aromatic nucleus and oxygen-containing groups [38], in which the oxygen-containing groups decorated on the basal planes and edges are responsible for hydrophilicity and uniform dispersion in water [37,39]. As the reduction process goes on, local phase separation will occur, caused by the removal of hydrophilic groups, which further generates the deformation to prepare for self-assembly. Furthermore, the hydrogen bonding reaction between residual hydrophilic regions of the reduced GO and water causes adsorption of some water drops on the surface, serving as a spacer to prevent parallel aggregation of nanosheets [40].

**Figure 3:**
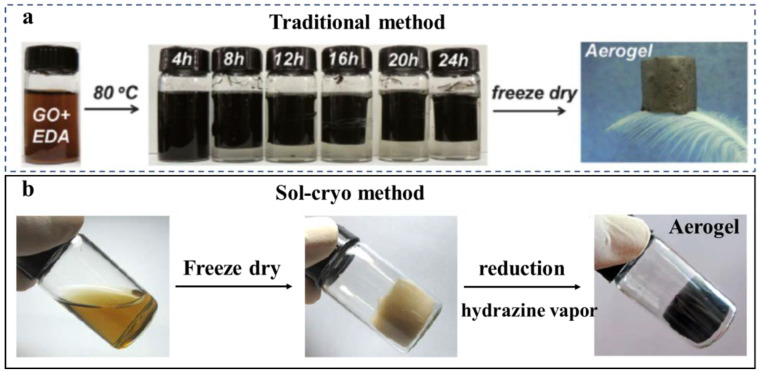
(**a**) Images of the fabrication process of the graphene aerogel using the traditional method, where the GO is reacted with EDA for 24 h at 80 °C and then freeze-dried. Reprinted with permission from Ref. [44]. Copyright 2014, The Royal Society of Chemistry. (**b**) Images of the fabrication process of the graphene aerogel using the sol-cryo method. Reprinted with permission from Ref. [46]. Copyright 2013, Wiley-VCH.

**Chemical reduction** is the most widely used method to realize the transformation from GO to graphene. The common reducing agents include ethylenediamine (EDA), hydrazine vapor, Fe (II), sodium hydrogen sulfite (NaHSO_3_), sodium sulfide (Na_2_S), and vitamin C and hydrogen iodide (HI) [23,41]. The traditional chemical reduction method to prepare graphene aerogels involves three processes: (i) reduction of graphene oxide aqueous solution, (ii) self-assembly of graphene sheets to form hydrogel, and (iii) lyophilization of hydrogel. Inspired by a mild method to synthesize 3D architectures of graphene [23,42], Yan et al. [43] prepared the magnetic 3D graphene/Fe_3_O_4_ aerogel via in situ self-assembly of graphene with NaHSO_3_ as the reducing agent in the presence of Fe_3_O_4_ nanoparticles. The capture of nanoparticles was similar to a fishing process, wherein nanoparticles acted as the fish and graphene/GO nanosheets served as the fishing net that was formed through the self-assembly of GO during reduction. Similarly, Li et al. [44] proposed a simple method, which referred to a one-step reduction by EDA and the self-assembly of graphene oxide, and then freeze-drying to prepare porous graphene aerogel, as shown in Figure 3a. In order to eliminate residual functional groups to obtain ultralight aerogel, Qiu et al. [45], moving one step further, designed a two-stage process. GO was controllably functionalized and assembled into monolithic graphene hydrogel by introducing ethylenediamine. After freeze-drying, the obtained aerogel was subjected to microwave irradiation treatment to eliminate functional groups. Furthermore, Gao et al. [46] developed a novel sol-cryo method to fabricate ultra-flyweight carbon aerogels (Figure 3b). Compared to the traditional approach prepared from hydrogels, they cryodesiccated the aqueous CNTs and giant GO sheets rather than the hydrogels, followed by chemical reduction with hydrazine vapor. 

**Figure 4:**
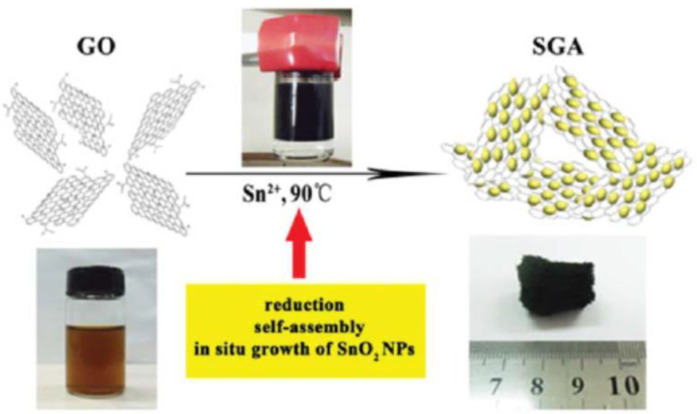
A schematic illustration of the synthesis of the SGA. Reprinted with permission from Ref. [50]. Copyright 2013, The Royal Society of Chemistry.

**Hydrothermal reduction** is another facile and effective strategy to prepare graphene from GO. The reduction degree is determined by reaction temperature and time. Generally speaking, the higher the temperature, the higher the reduction degree. However, there is no consensus on the required time and temperature of the heat-treatment for complete reduction of GO [47,48]. Most of the hydrothermal reduction induced self-assembly of 3D graphene aerogel occurs in the range 80–150 °C [49]. Li and colleagues [50] proposed a facile one-step method to prepare 3D macroscopic SnO_2_-graphene aerogel (SGA), which involved the synchronous processes of hydrothermal-induced reduction of GO, self-assembly of graphene sheets, and in suit growth of SnO_2_ nanoparticles, presented in Figure 4. Similarly, Niu et al. [51] reported a universal strategy to incorporate various nanomaterials into 3D aerogel: dispersion of GO and functional components under ultrasonication, followed by heat-treatment at 180 °C and freeze-drying of the hydrogel. 

**Figure 5:**
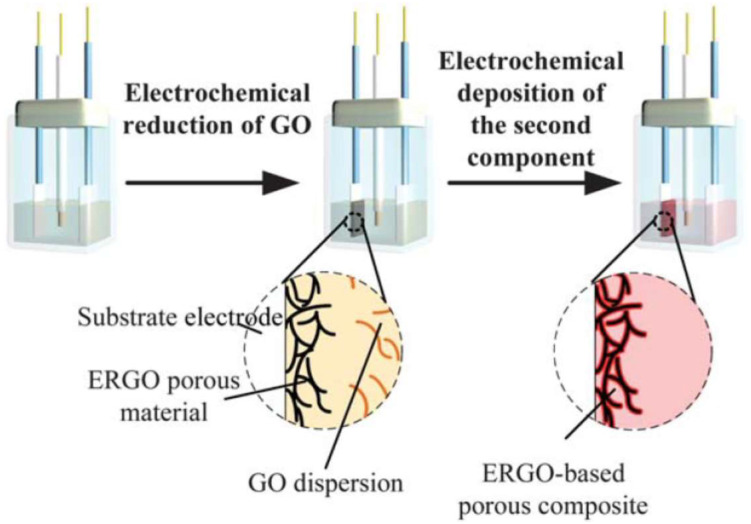
Schematic illustration of the preparation method for ERGO-based composite materials. Reprinted with permission from Ref. [24]. Copyright 2012, The Royal Society of Chemistry.

**Electrochemical reduction** is an appealing strategy used to form 3D GA. Unlike other self-assembly methods that rely on the unique shape of graphene sheets and features of the second components and whose materials are bulky monoliths and unsuitable for electrode modification, electrochemical reduction-induced self-assembly approach is believed to be the most promising means to fabricate a 3D structure applied in electrode material. Chen et al. [24] proposed a method consisting of two consecutive electrochemical steps, namely, electrochemical reduction of GO in aqueous dispersion followed by electrochemical deposition of the second components to form 3D graphene-based composite materials, as shown in Figure 5.

#### 2.1.2. Crosslinking Induced Self-Assembly

The abundant oxygen-containing functional groups on their basal planes and edges make GO sheets sensitive to the experimental conditions, and only assemble into 3D microstructure under certain conditions [52]. The obtained 3D aerogels without reinforcement usually have poor mechanical properties [53]. Therefore, adding cross-linking agents such as polymers [54,55], small organic molecules [56], biomacromolecules [57], and multivalent ions or nanoparticles [58] during synthesis procedures is an effective way to improve their mechanical properties and ordered organization.

**Figure 6:**
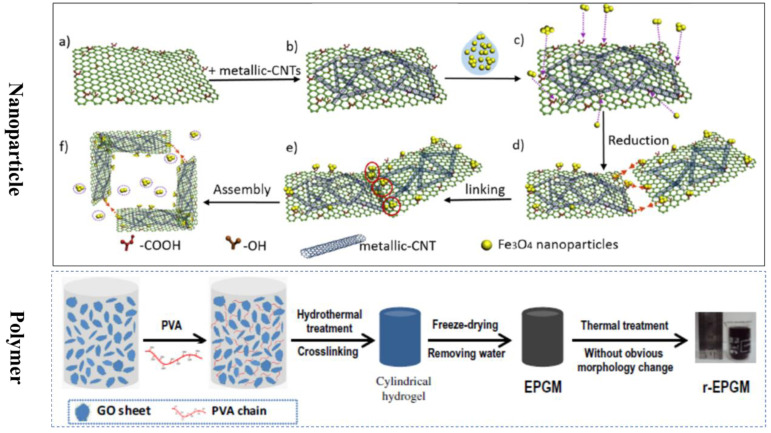
Schematic diagram for the preparation procedure and mechanism of CGA; (**a**) GO flakes; (**b**) metallic-CNT on GO flakes; (**c**) Fe_3_O_4_ NPs on the edge of GO flakes; (**d**) Fe_3_O_4_ NPs attracted with each other; (**e**) Fe_3_O_4_ NPs enhanced the interlaminar connectivity of flakes; (**f**) the redundant NPs on the pore of CGA. Reprinted with permission from Ref. [58]. Copyright 2019, American Chemical Society. Schematic showing the procedure for the preparation of r-EPGM. Reprinted with permission from Ref. [55]. Copyright 2013, Elsevier Ltd.

**Physical cross-linking** refers to linking GO sheets and a crosslinker mainly by hydrogen bonding, as illustrated in Figure 6. Qin and co-workers [58] fabricated a reinforced composite graphene aerogel (CGA) by combining surface support through metallic-carbon nanotubes (CNTs) networks and interfacial cross-linking of GO sheets with magnetic nanoparticles (NPs) by a hydrogen-bond. The continuous network structure contributes to mechanical stability and preferred adsorption sites of magnetic NPs. Polymers, such as poly(vinyl alcohol) (PVA), chitosan (CS), cellulose, and poly(N-iso-propylacrylamide) are also employed to act as a crosslinker. Tao et al. [55] crosslinked the functionalized GO sheets with PVA under hydrothermal conditions to prepare reduced expanded porous graphene macroform (r-EPGM) aerogel, whose pores range from micropores to mesopores to macropores. Similarly, Asfaram and coworkers [59] successfully fabricated a novel 3D magnetic GO composite aerogel depending on the hydrogen bond between the polymer crosslinking agent (PVA) and other components (bacterial cellulose nanofibers, Fe_3_O_4_ nanoparticles and GO nano-sheets).

**Figure 7:**
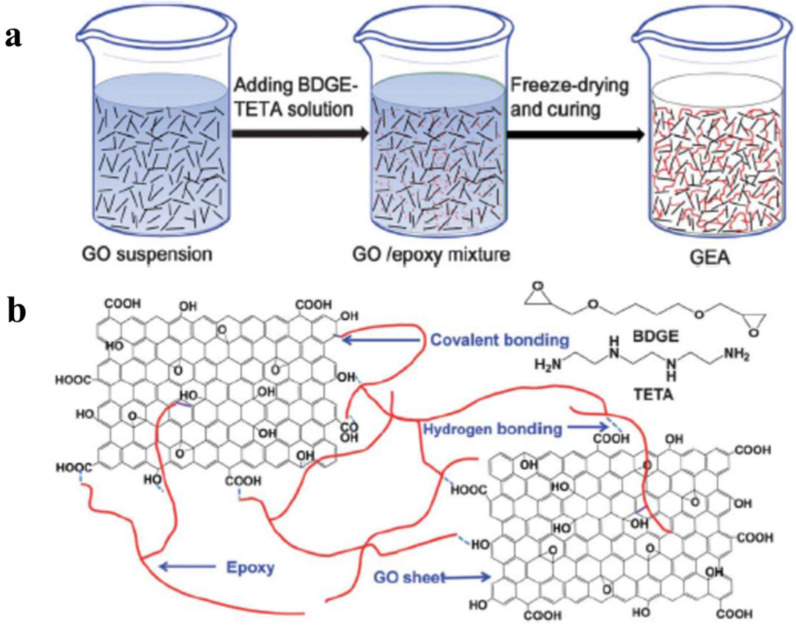
(**a**,**b**) Refer to the scheme of the preparation and formation mechanism of GEAs, respectively. Reprinted with permission from Ref. [56]. Copyright 2013, The Royal Society of Chemistry.

**Chemical cross-linking** refers to linking GO sheets and a crosslinker by covalent bonding, which has the characteristics of high uniformity and stabilization. Ye et al. [56] developed a facile two-step method consisting of freeze-drying and subsequent curing process to generate inter-crosslinked GO-epoxy composite aerogels (GEAs) with epoxy as a crosslinkable polymer and mainly through covalent bonding interaction (Figure 7). The resulting GEAs exhibit excellent compressive strength, high elasticity, and thermal stability, allowing them to be applied in energy absorption and durable insulation materials.

During the synthesis process, hydrogen bonding and/or covalent bonding between crosslinker and GO sheets accounts for forming the cross-linking sites, which contribute to the generation of hydrogel with a 3D network. Such aerogels usually show significantly improved mechanical properties. However, the introduction of cross-linkers inevitably sacrifices the electrical conductivity of GAs to a certain extent. So, there is a fundamental tradeoff to grasp. Utilizing conductive polymer as a crosslinker to fabricate highly compressible and electrically conductive 3DGAs might be a new attempt.

### 2.2. Template-Directed Approach 

The template-directed strategy, including the emulsion technique, breath figure method, and ink-printing technique, is one of the mainstream approaches employed in the formation of 3D GA with ordered and hierarchical structures [60]. It uses a pre-existing guide (hard or soft template) to directly synthesize the target materials that are difficult to obtain by other tools. Meanwhile, it also inevitably limits the scalability of resulting aerogels owing to the hard accessibility of a well-organized and large-size template itself [46]. The general route for a templated-directed method mainly involves the following steps: (1) template preparation, (2) synthesis of target materials using the template, (3) template removal (if necessary). The obtained aerogels were widely used in lithium-ion batteries and proved to be excellent electrode materials with outstanding performance.

#### 2.2.1. Hard Template

**Figure 8:**
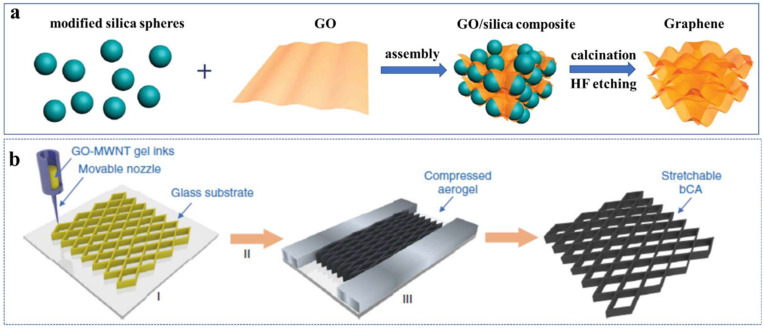
(**a**) Schematic illustration of the synthesis procedures of the nanoporous graphene foams. Reproduced with permission from Ref. [63]. Copyright 2012, Wiley-VCH. (**b**) Schematic illustration of the hierarchical synergistic assembly for fabrication of stretchable bCAs through 3D printing (I) followed by freeze-drying (II) and pre-buckled reduction (III). Reprinted with permission from Ref. [31].

Hard templating strategy, using solid nanoparticles, porous metal foams, and specific movable nozzles as a guide is conceptually the simplest method to synthesize 3D graphene aerogel with the desired microstructure and performance [61]. In general, the pore size is highly determined by the template, and the templates usually need to be removed completely by physical or chemical methods [62]. Huang et al. [63] prepared nano-porous graphene foams by using methyl group modified silica spheres as the hard template. They mixed the silica spheres and GOs in a neutral aqueous to form self-assembly, calcined the obtained composite to reduce GOs into graphene, and etched silicas with HF to produce nanopores. In addition, Guo and coworkers [31] utilized an intelligent 3D ink-printing technique that involves three procedures: ink-printing aqueous of GO and multiwalled carbon nanotubes (MWNTs) with trace calcium ions as the gelator, followed by freeze-drying and reduction under a confined state to fabricate highly stretchable graphene/MWNTs composite aerogel (bCA) with four orders of hierarchical structures (Figure 8).

#### 2.2.2. Soft Template

Compared with the hard template approach, the soft template method with the emulsion droplet, vesicle/micelle, and gas bubble as templates is easier due to the unnecessity of removing the templates [64]. Although it offers less control over the uniformity of the obtained structures, it offers more possibilities in tuning and producing complicated hierarchical structures [65], which has been widely used to prepare 3D graphene porous architectures. Huang et al. [28] employed multiple microemulsions and micelles as a soft template to synthesize 3D porous graphene foams with tunable pore structures. Sun and co-workers [66] proposed a breath-figure method to macro-porous graphene films, by virtue of condensation and close packing driven by the evaporation of the volatile organic solvent of humid air, which promotes the self-assembly of GO platelets. The subsequent drying resulted in a porous film. However, there have been no reports on the fabrication of real graphene aerogels using the soft template method.

The carbon aerogels (CNTs, GO, and graphene) prepared by various methods generally have lower density than the graphene/GO-based hybrid (other nanoparticle or polymers) aerogels. Moreover, for the same GO aerogels obtained by the two-stage preparation procedure involving subsequent microwave irradiation (MWI) or thermal treatment, which further eliminates functional groups after freeze-drying, are ultralight and their density came down to a lower scale compared with those prepared via one-step. However, in any case, all the as-prepared GA possess an interaction network and hierarchical pores ranging from micropores to mesopores and macropores, which can be characterized by SEM and the nitrogen adsorption/desorption isotherm [67]. The porosity is usually higher than 99.6%. The porous structure contributes to a high specific surface area and/or porosity and provides a material transport channel and accessibility to the active surfaces, making them a promising candidate as an adsorbent of organic liquids, anode material, and energy adsorption and storage material. The porous features, surface aera, and density of typical GAs prepared by various methods are summarized in Table 1.

## 3. Application

Graphene aerogel, with characteristics of low density, high surface area and porosity, and good electrical and thermal conductivity, has attracted the attention of researchers and flourished in the recent decades [68]. They possesses potential application in diverse fields, such as sorption in environmental protection [26,69] electrode materials [70,71], electronic devices [72], flame-retardant and fire-warning materials [73,74], catalysis [75], energy storage [76], and microwave absorption [58]. Based on the adsorption, electrochemistry, mechanical, catalytic, and fire-warning properties of GAs, here we mainly summarize its application fields and classify it into five aspects (absorbent, anode material, mechanical device, fire-warning materia,l and catalyst) sequentially.

### 3.1. Aerogels for Absorption

Water contamination caused by harmful chemicals, particularly oils and soluble dyes and phosphate, has become an issue of serious global concern. Various technologies including chemical precipitation [77,78], biological treatment [79], membrane filtration [80,81], adsorption [82], and ion exchange [83] have been employed to remove organic contaminants from wastewater. Among them, the adsorption method has been recognized as the most promising candidate. Therefore, the preparation of novel absorbents with low density, water pickup and cost, high absorption capacity, and good recyclability is in urgent need.

**Figure 9:**
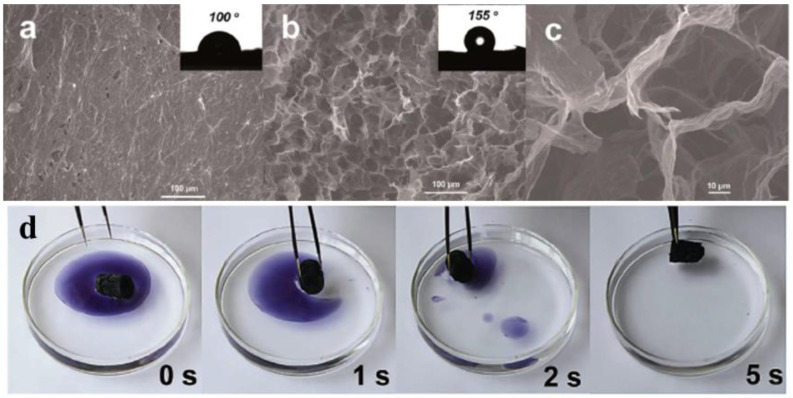
SEM images of the (**a**) surface, (**b**,**c**) cross-section of the graphene aerogel showing the porous network structure. Reprinted with permission from Ref. [44]. Copyright 2014, The Royal Society of Chemistry. (**d**) Absorption process of toluene (stained with Sudan Black B) on water by the aerogel within 5 s. Reprinted with permission from Ref. [46]. Copyright 2013, Wiley-VCH.

Due to the super-hydrophobicity of GA [84], it is commonly considered as a competitive and efficient adsorbent for oil in water, with a higher adsorption capacity compared to other kinds of adsorbents. Li et al. [44] prepared the porous graphene aerogel by one-step reduction and self-assembly of GO. The aerogel is super-hydrophobic with a contact angle of 155° and is an ideal candidate for oil absorption (Figure 9a–c). Taking *n*-decane adsorption as an example, 8.5 mg of aerogel can completely adsorb 1.4 g of oil within 6 s. The average absorption rate (27 g/g·s) is much faster than that of pure graphene (0.57 g/g·s). The capacity for absorption is up to 120–250 g/g, depending on the liquid densitY.S.milarly, Gao et al. [46] fabricated the ultra-flyweight aerogels by virtue of sol-cryo methodology, which shows excellent absorption capacity of extensive oil liquids (such as *n*-hexane, crude oil, toluene, motor oil, vegetable oil, ionic liquid, and phenixin) (Figure 9d), falling into the range of 215–913 times their own weight dependent on the oil density, which is 1–2 orders of magnitude higher than traditional adsorbents.

**Figure 10:**
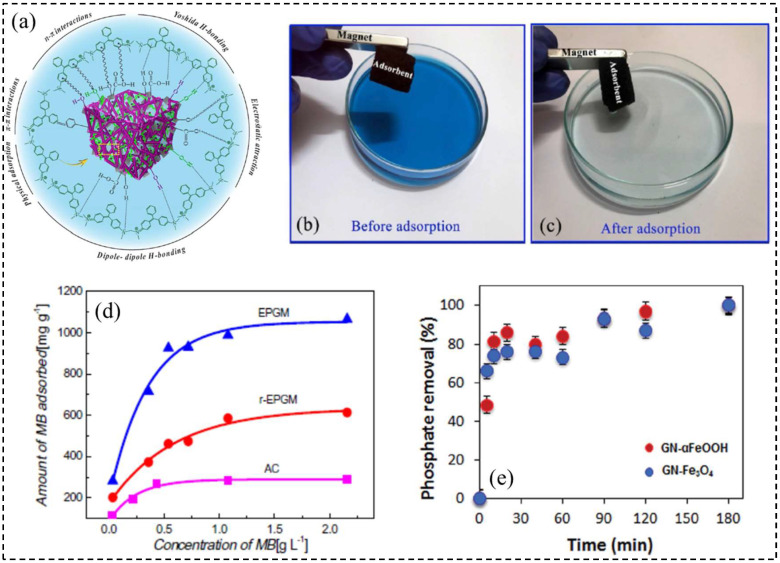
(**a**) Schematic illustration of the possible interaction between aerogel and MG dye; (**b**,**c**) images of adsorption properties and magnetic separation of aerogel before and after the MG dye adsorption process. Reprinted with permission from Ref. [59]. Copyright 2019, Elsevier B.V. (**d**) Adsorption isotherms of r-EPGM, EPGM and AC toward MB at 303 K and pH = 11.5. Reprinted with permission from Ref. [55]. Copyright 2013, Elsevier Ltd. (**e**) The effect of time on the amount of phosphate adsorbed on the GN-αFeOOH and GN-Fe_3_O_4_ aerogels. Conditions: phosphate concentration = 20 mg/L; pH = 6.0. Reprinted with permission from Ref. [85]. Copyright 2015, The Royal Society of Chemistry.

Taking advantage of the abundant oxygen-containing groups and unique pore structure, GO aerogels also exhibit excellent adsorption capacity of water-soluble dye and phosphate pollutants. Tao and co-workers [55] prepared expanded porous graphene macroform (EPGM) aerogel by crosslinking the functionalized GO sheets with PVA under hydrothermal conditions. It has superior adsorption capacity (1050 mg/g) for methylene blue (MB) dye, which is much higher than the typical microporous carbon (AC) (Figure 10d). Asfaram and coworkers [59] successfully fabricated a novel 3D magnetic GO composite aerogel via a gentle filler-loaded networks method. The aerogel is characterized by, e.g., an interconnected porous structure, lightweight (6.8 mg/cm^3^), high surface area (214.75 m^2^/g), paramagnetic property (26.59 emu/g), excellent adsorbent efficiency (93%) for cationic malachite green (MG) dye through the Yoshida H-bonding, dipole-dipole H-bonding, π-π interaction, *n*-π interaction, electrostatic attraction, and physical adsorption, as shown in Figure 10a,c. The result showed that the aerogel preserved a maximum adsorption capacity of 270.27 mg/g for MG. According to previous research, metal oxide with high surface area and affinity for the phosphate, especially iron sulphate (FeSO_4_), once adhered to the graphene or GO, will show great application potential in capturing phosphates in water, Losic et al. [85] proposed a facile and green method to fabricate two types of 3D graphene aerogels embedded αFeOOH and Fe_3_O_4_ nanoparticles, respectively. The prepared aerogels showed superior capacity of 350 mg/g at an initial phosphate concentration of 200 mg/L under acidic conditions and the adsorption process followed the second order model and Freundlich isotherm (Figure 10e).

GAs with unique microstructure act as an ideal guide, providing a new probability to solve the serious water pollution problem faced by traditional adsorbents. The improved adsorption performance, lower-cost, and expanded application in contaminant removal of the GAs is expected to be achieved in the future.

### 3.2. Aerogels for Anode Material of Rechargeable Lithium-Ion Batteries

Rechargeable lithium-ion batteries (LIBs) with high energy density and voltage, which can store and supply electricity, have a wide range of applications with the development of modern electronic devices. Currently, the biggest challenge is to develop durable, nontoxic, and inexpensive materials for electrodes.

**Figure 11:**
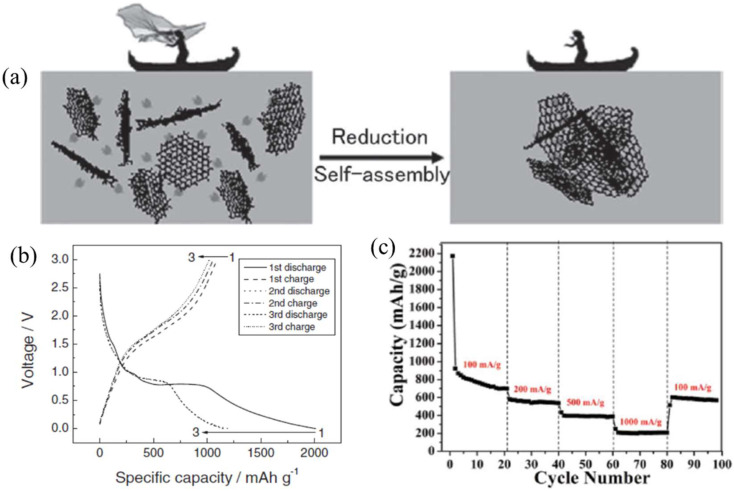
(**a**) The proposed self-assembly and wrapping mechanism for 3D graphene/nanoparticles architecture formation during the chemical reduction of GO in aqueous suspension in the presence of nanoparticles. (**b**) Electrochemical characterizations of a half-cell composed of graphene/Fe_3_O_4_ and Li: discharge/charge profiles. Reprinted with permission from Ref. [43]. Copyright 2011, Wiley-VCH. (**c**) Rate performance of the SGA. Reprinted with permission from Ref. [50]. Copyright 2013, The Royal Society of Chemistry.

Graphene, the single layer of carbon atoms in a hexagonal lattice, is endowed with high stability and unique electronic properties [86], such as conductivity and carrier mobility. Theoretically, transition metal oxide has high theoretical specific capacity, so transition metal oxide/graphene composite aerogels are promising candidates for anode materials of lithium-ion battery and have attracted the most attention of researchers. The monobasic transition metals of Mn_3_O_4_ [87], Co_3_O_4_ [86], Fe_3_O_4_ [88,89], and SnO_2_ [50] and the binary transition metal of ZnFe_2_O_4_ [90], combined with graphene or reduced GO, have been studied for anode materials. Yan et al. [43] prepared a magnetic 3D graphene/Fe_3_O_4_ aerogel via in situ self-assembly of graphene in the presence of Fe_3_O_4_ nanoparticles. The graphene/Fe_3_O_4_ composite aerogel showed a high capacity and cyclic stability (a remaining capacity of 1100 mA h/g after 50 cycles of charge and discharge), meaning it can work well as an anode for LIBs, as shown in Figure 11a,b. The excellent performance is attributed to the efficient interaction between Fe_3_O_4_ nanoparticles working as spacer and graphene nanosheets, as both sides can adsorb lithium ions [91]. Similarly, Li and colleagues [50] proposed a facile one-step method to prepare 3D macroscopic SnO_2_-graphene aerogel (SGA), which displays a well-defined and interconnected network porous microstructure and possesses higher capacity retention and discharge capacity (602 mA h/g after 60 cycles) compared to other SG composites (Figure 11c). Moreover, it can also tolerate varied discharge current densities and maintain a relatively high specific discharge capacity of 590 mA h/g (about 64% of the initial reversible capacity). These research works have created a foundation for wide applications of novel 3D graphene/nanoparticle aerogels in the future.

Owing to the synergetic effect of the super-flexible coating provided by graphene nanosheets and reversible Li^+^ storage capacity by metal oxide nanoparticles, the composite aerogel has improved performance more suitable for the anode material of LIBs. The high surface area and continuous porous structure of graphene aerogel is attributed to its superior specific capacitance, making it attractive as advanced electrode materials.

### 3.3. Aerogels with Mechanical Stability for Novel Devices

Two-dimensional graphene/GO with outstanding tensile and compressive strength, high flexibility, and elasticity [92,93] is commonly considered as the most promising building block to fabricate 3D aerogel with mechanical stability. Such an aerogel can be widely employed in flexible electronics, sensors, wearable devices, and smart manufacturing [31]. However, monolithic graphene aerogels formed by random assembly of graphene sheets directly via weak connection often exhibit obvious brittleness in compression as well as stretch [94], and has difficulties meeting the application demands.

**Figure 12:**
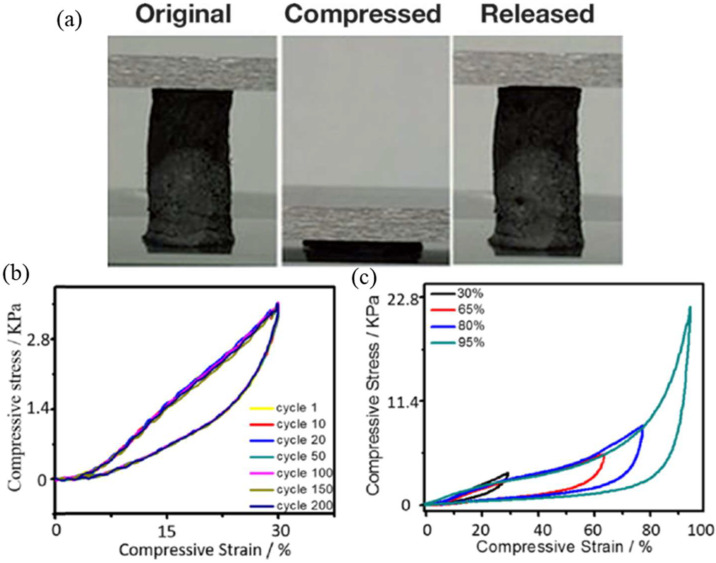
(**a**) Digital photographs showing the compressibility of aerogels. Reprinted with permission from Ref. [45]. Copyright 2013, Wiley-VCH. (**b**) Stress–strain curve of CGA at the maximum strain of 30% for 1–200 cycles; (**c**) stress–strain curve of CGA at different maximum strains of 30, 65, 80, and 95% respectively. Reprinted with permission from Ref. [58]. Copyright 2019, American Chemical Society.

There are two main strategies to overcome the brittleness to pursue 3D aerogels with mechanical robustness. The predominant approach is to introduce elastic polymers and small molecules acting as cross-linkers or barriers into the matrix [45,58,95,96], which is less stable in severe chemical or physical conditions [97]. On the basis of graphene aerogel preparation regarding reduction, Qiu et al. [45] designed an integrated functionalization and assembly with the following microwave irradiation treatment process to fabricate ultralight aerogels with high compressibility. It can recover from strain as high as 90% when squeezed into a flake without obvious variation in volume, as shown in Figure 12a. Its properties are beneficial to potential applications in the fields of shock damping and energy absorption. By combining the surface support through metallic-CNTs networks and interfacial cross-linking of GO sheets with NPs, Li et al. [58] fabricated a reinforced composite graphene aerogel (CGA) with excellent structural stability and elastic deformation performance, which show that the stress−strain curves remain unchanged after 200 cycles under the maximum strain of 30% and recover to the origin point at the strain up to 95% (Figure 12b,c).

**Figure 13:**
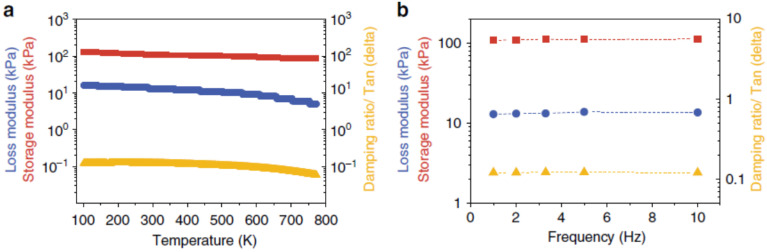
Mechanical stability of bCAs. Storage modulus (red), loss modulus (blue), and damping ratio (yellow) as a function of temperature (**a**) and frequency (**b**) of bCAs with 30 wt% MWNTs. Reprinted with permission from Ref. [31].

Another approach is to enhance the interconnection of aerogels to produce a hierarchical structure by adopting freeze-shaping, 3D ink-printing, and other synergistic assembly techniques [98,99]. Guo and coworkers [31] adopted an intelligent 3D ink-printing technique to fabricate highly stretchable graphene/multiwalled carbon nanotubes (MWNTs) composite aerogel (bCA) with four orders of hierarchical structures, which makes a great contribution to the superior mechanical performance of bCA, including low energy dissipation (0.1, 100% strain), high fatigue resistance (more than 106 cycles), minor plastic deformation (1%), and excellent environment stability (93–773 K), as shown in Figure 13. The ultralight CAs (9.7 mg/cm^3^), with mechanical stability under dynamic tension and compression deformations, can find potential applications in strain sensors, stretchable components, and lightweight mechanical devices.

Based on the major advances made in the design of flexible 3D graphene aerogels, the trend is to push their structure towards a smarter and more well-controlled system [100], in order to fully understand the nature of property degradation during two-dimensional individual assemblies into bulk 3D structures, and to promote advances in more intelligent design and extensive application in flexible electronic devices, sensors, and complex mechanical structures. 

### 3.4. Aerogels for Fire-Warning Material

The existing commercial fire-warning equipment, including temperature, smoke, and infrared flame detectors, is usually unsatisfactory [101], as they are commonly located at a certain distance from the combustion source and are triggered only when the smoke concentration or temperature reaches a critical value [102]. Consequently, the fire-warning is insensitive, with a response time of more than 100 s [103], which is too late to curb the fire spread and misses the best time for evacuation.

**Figure 14:**
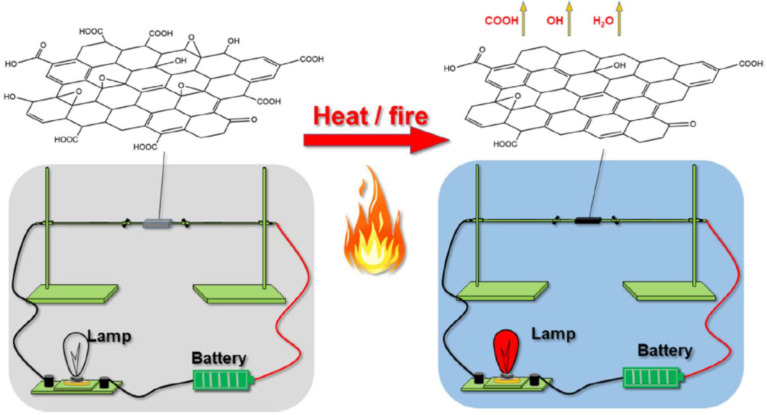
Experimental circuit and fire-warning mechanism diagram. Reprinted with permission from Ref. [74]. Copyright 2021, John Wiley & Sons Ltd.

With the increase of temperature, the electrical resistance of GO decreases dramatically, which endows it an attractive application prospect in fire-warning materials [101,104,105]. However, because of its unique porous network structure, aerogel inevitably encounters difficulties in reducing electrical resistance during being burned, which remains a challenge to fabricate sensitive fire-warning aerogels [106,107]. Yuan et al. [73] creatively prepared the GO/ammonium polyphosphate/cellulose nanofiber composite aerogel with a fire-response time of 2.6 s through freeze-drying for the first time. The aerogel exhibits excellent thermal-isolating, flame-retardant, and timely fire-alarm properties. Furthermore, they [74] also fabricated a GO/sodium montmorillonite/cellulose nanofiber multifunctional composite aerogel that triggered a fire alarm in about 1.9 s when met with a fire. Owing to the thermal reduction characteristic of GO, referring that quickly removes oxygen-containing groups, and being reduced to graphene once encountering high temperature or fire, the aerogel possesses abilities of timely detection and early warning in the pre-combustion stage, as illustrated in Figure 14.

The GO modified aerogel with excellent flame retardancy, thermal isolation, and intrinsic fire warning performance broadens its application territory to cover the drawbacks in delayed response and restricted application scenarios of traditional fire detectors. Therefore, the aerogel that can be triggered in the precombustion stage to offer favorable opportunities for firefighting and emergency rescue is endowed with enticing prospects in chemical industries, pipeline transportation, and high-rise buildings.

### 3.5. Aerogels for Catalysis

A 3D network structure provides multidimensional electron transport pathways and large accessible surface area, which is conducive to improve the separation efficiency of photogenerated electron-hole pairs and the adsorption of reactants. Such intrinsic hierarchical porous structure characteristics and properties make GAs endowed with potential as promising and efficient photocatalysts for practical applications in solar energy conversion [108], such as pollutant elimination [109], water splitting [110], CO_2_ reduction [111], and chemical reaction progress [112].

**Figure 15:**
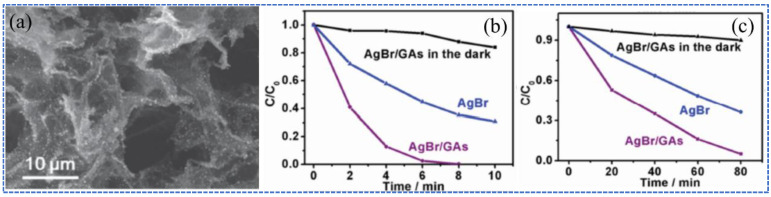
(**a**) SEM image of the AgBr/GAs. (**b**,**c**) The photocatalytic oxidative curves of MO and reductive curves of Cr^VI^ by AgBr/GAs and AgBr under visible light and the absorptive curve of AgBr/GAs in the dark, respectively. Reprinted with permission from Ref. [114]. Copyright 2016, Elsevier Inc.

The conventional photocatalysts in powder form used in photocatalytic pollutant decomposition has difficulties in meeting the needs of the recycling process [113], as it is essential to immobilize the photocatalyst on solid support. The monolithic 3D GAs is undoubtedly a desirable photocatalyst carrier. Fan et al. [114] prepared a novel 3D AgX/GA (X = Br, Cl) structure, in which the AgX NPs were uniformly distributed on the surface of GA by in situ growth method (Figure 15a). The GAs exhibited high catalytic properties in the oxidative degradation of methyl orange (MO) and the reduction of Cr^VI^, showing that after visible light irradiation for 8 min, the MO was completely degraded by AgBr/GAs compared with the 65% of degradation rate by AgBr (Figure 15b); and the reduction ability of Cr^VI^ was 1.5 times higher than that by bare AgBr at the same time interval (Figure 15c).

**Figure 16:**
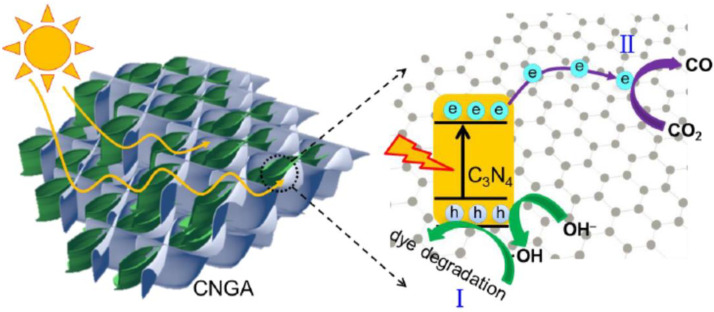
Schematic diagram for illustrating the photodegradation (I) and photoreduction (II) processes over CNGA under visible light irradiation. Reprinted with permission from Ref. [75]. Copyright 2015, American Chemical Society.

Generally, CO_2_ reduction usually undergoes two processes: oxidizing water to generate hydrogen ions (2H_2_O + 4h^+^→O_2_ + 4H^+^) and reducing CO_2_ to CH_4_ via acquiring 8-electrons process (CO_2_ + 8H^+^ + 8e^–^→CH_4_ + 2H_2_O) [115]. The 3D GAs have also been proved to be an exciting photocatalyst for the reduction of main greenhouse gas CO_2,_ with higher catalytic efficiency compared to other semiconductors. Tong and co-workers [75] designed a 3D porous g-C_3_N_4_/GO aerogel (CNGA) by the hydrothermal induced self-assembly method, in which g-C_3_N_4_ acted as the efficient photocatalyst, and GO was responsible for supporting the 3D framework. The as-prepared GAs could reduce CO_2_ into CO with a high yield of 23 mmol/g (within 6 h), which was about 2.3-fold increment compared to pure g-C_3_N_4_ (Figure 16). That is because the photogenerated electrons transfer to the network of GO and react with the CO_2_ molecules under visible light irradiation. The holes that remained in the valence band of g-C_3_N_4_ can react with the surface adsorbed H_2_O. The separated electron-hole pairs fix the oxidative and reductive reactions mainly on the g-C_3_N_4_ and GO surface, leading to highly efficient photocatalytic performance.

The 3D GAs also play an important role in catalyzing some chemical reactions, such as the selective oxidation of alcohol to carbonyl, the reduction of nitroaromatic compound to amino compound, and the synthesis of ammonia [65]. Yang et al. [111] prepared a metal-free 3D graphene-organic aerogel, in which the organic dyes acted as photosensitizers. Such graphene-dye aerogels showed high photocatalytic activity for the hydrogenation of nitro compounds to amines and the reduction of heavy metal ions, which was calculated to be 1.4 and 1.8 times as high as those of other semiconductor-organic dye materials, respectively.

## 4. Future Perspectives

There is no doubt that considerable advances have been achieved in the fabrication of 3D GA materials in the past few decades. Various preparation methods involving a series of template-free and template-guided methods are proposed to fabricate 3D GAs with integrated properties of graphene and other unique characters. The excellent properties make them attractive and promising in potential applications in varied fields, which brings us closer to practical applications. In this review, we summarized the advancement of literature, historical progress of synthesis of 3D GAs, and introduced the main application in absorption, anode material, mechanical device, fire-warning material, and catalysis aspects.

Currently, although dramatic progress has been realized, the performance and application filed of 3D GAs has not been fully exploited. There are still many challenges toward the preparation and mechanism elaboration of highly efficient 3D GAs. Firstly, most of the 3D GAs are derived from the reduction of GO, so their electrical conductivity and charge carrier mobility are remarkably decreased due to the disruption of *p*-conjugation compared to pure graphene, which causes the improvement of electrical correlation performance of 3D GAs to often be restricted. Therefore, rational utilization of graphene with superior electrical conductivity to advance the 3D GAs still needs continuous efforts. Secondly, the widely used drying methods of preparation of aerogel are mainly freeze-drying and supercritical drying [116]. Freeze-drying has the principal shortcomings of high energy consumption, prolonged processing time, and microcrystals formation. Supercritical drying gives aerogel a 3D-porous structure with small pores similar to wet gel, which faces limited development due to a complex process and expensive equipment [117]. Therefore, the advent of non-supercritical drying techniques may further expand the aerogels field in a near future. Thirdly, the research of 3D GAs for fire-safety material is still in its elementary stage, and the mechanism and characterization means of fire-warning response need further improvement [105]. Therefore, deeper systematic research on the mechanism from theoretical and experimental aspects is highly desired.

With the output of constant efforts, we believe that substantial breakthroughs for the finer structural control and practical applications of 3D GAs would be expected in the near future.

## Figures and Tables

**Table 1:** Porous features, surface aera, and density of typical GAs prepared by various methods.

Aerogel	Synthesis Methods	Reaction Condition	Density (mg/cm^3^)	Porous Features	Surface Aera/Porosity	Application	Ref.
Graphene /Fe_3_O_4_	Chemical reduction	95 °C/3 h Nahso_3_One-step	42	Macropores Mesopores	95.22 m^2^/g	Anode material	39
GO	Chemical reduction	95 °C/6 h EDA Two-stage	3	Macropores	99.7–99.8%	Shock damping/ Energy absorption	41
GO	Chemical reduction	80 °C/24 h EDA One-step	4.4–7.9	Macropores	99.6%	Organic absorbent	40
CNTs/GO	Chemical reduction	90 °C/24 h N_2_H_4_ Sol-cryo One-step	0.16–22.4	Macropores	99.9%	Organic absorbent Conductor	42
SnO_2_– graphene	Hydrothermal reduction	90 °C/1 h One-step	/	Macropores	/	Anode material	46
GHAs	Hydrothermal reduction	180 °C/3 h	/	Macropores	/	Electrode material	47
ERGO	Electrochemical reduction	/	/	Macropores	1000 m^2^/g	Electrode material	22
CGA	Cross-linking	140 °C/2 h Two-stage	11.1	Macropores	/	Microwave absorption material	54
r-EPGM	Cross-linking	150 °C/6 h Two-stage	/	Micropores mesopores Macropores	>1000 m^2^/g	Adsorbent/ Energy storage material	51
GEA	Cross-linking	100 °C/24 h One-strp	90	Macropores	/	Energy absorbing/ Durable insulation material	52
CNTs/GO	Template	Two-stage	5.7	Macropores	/	Strain sensor	29

## References

[1] Kistler S.S. (1931). Coherent expanded aerogels and jellies. Nature.

[2] Yu M.C., Zhang H.M., Yang F.L. (2017). Hydrophilic and compressible aerogel: A novel draw agent in forward osmosis. ACS Appl. Mater. Inter..

[3] Antonietti M., Fechler N., Fellinger T.-P. (2013). Carbon aerogels and monoliths: Control of porosity and nanoarchitecture via sol-gel routes. Chem. Mater..

[4] Pierre A.C., Pajonk G.M. (2002). Chemistry of aerogels and their applications. Chem. Rev..

[5] Ulker Z., Erkey C. (2014). An emerging platform for drug delivery: Aerogel based systems. J. Control. Release.

[6] Ziegler C., Wolf A., Liu W., Herrmann A.K., Gaponik N., Eychmuller A. (2017). Modern Inorganic Aerogels. Angew. Chem. Int. Ed. Engl..

[7] Liu Z.J., Ran Y.Y., Xi J.N., Wang J. (2020). Polymeric hybrid aerogels and their biomedical applications. Soft Matter.

[8] Stergar J., Maver U. (2016). Review of aerogel-based materials in biomedical applications. J. Sol-Gel Sci. Techn..

[9] Wang Y., Guo L., Qi P.F., Liu X.M., Wei G. (2019). Synthesis of three-dimensional graphene-based hybrid materials for water purification: A review. Nanomaterials.

[10] Xiong C.Y., Li B.B., Lin X., Liu H.G., Xu Y.J., Mao J.J., Duan C., Li T.H., Ni Y.H. (2019). The recent progress on three-dimensional porous graphene-based hybrid structure for supercapacitor. Compos. Part B Eng..

[11] Yan Z.Q., Yao W.L., Hu L., Liu D.D., Wang C.D., Lee C.S. (2015). Progress in the preparation and application of three-dimensional graphene-based porous nanocomposites. Nanoscale.

[12] Li G.Y., Zhang X.T., Wang J., Fang J.H. (2016). From anisotropic graphene aerogels to electron- and photo-driven phase change composites. J. Mater. Chem. A.

[13] Hu K.W., Szkopek T., Cerruti M. (2017). Tuning the aggregation of graphene oxide dispersions to synthesize elastic, low density graphene aerogels. J. Mater. Chem. A.

[14] Kim C.H.J., Zhao D.D., Lee G., Liu J. (2016). Strong, machinable carbon aerogels for high performance supercapacitors. Adv. Funct. Mater..

[15] Lai F.L., Miao Y.-E., Zuo L.Z., Zhang Y.F., Liu T.X. (2016). Carbon aerogels derived from bacterial cellulose/polyimide composites as versatile adsorbents and supercapacitor electrodes. Chem. Nano. Mat..

[16] Kim K.H., Oh Y., Islam M.F. (2013). Mechanical and thermal management characteristics of ultrahigh surface area single-walled carbon nanotube aerogels. Adv. Funct. Mater..

[17] Korkmaz S., Kariper İ.A. (2020). Graphene and graphene oxide based aerogels: Synthesis, characteristics and supercapacitor applications. J. Energy Storage.

[18] Dong Q.C., Xiao M., Chu Z.Y., Li G.C., Zhang Y. (2021). Recent progress of toxic gas sensors based on 3D graphene frameworks. Sensors.

[19] Franco P., Cardea S., Tabernero A., De Marco I. (2021). Porous aerogels and adsorption of pollutants from water and air: A review. Molecules.

[20] Ibarra Torres C.E., Serrano Quezada T.E., Kharissova O.V., Kharisov B.I., Gómez de la Fuente M.I. (2021). Carbon-based aerogels and xerogels: Synthesis, properties, oil sorption capacities, and DFT simulations. Environ. Chem. Eng..

[21] Xu Y.X., Sheng K.X., Li C., Shi G.Q. (2010). Self-assembled graphene hydrogel via a one-step hydrothermal process. ACS Nano.

[22] Zhang X.T., Sui Z.Y., Xu B., Yue S.F., Luo Y.J., Zhan W.C., Liu B. (2011). Mechanically strong and highly conductive graphene aerogel and its use as electrodes for electrochemical power sources. J. Mater. Chem..

[23] Chen W.F., Yan L.F. (2011). In situ self-assembly of mild chemical reduction graphene for three-dimensional architectures. Nanoscale.

[24] Chen K.W., Chen L.B., Chen Y.Q., Bai H., Li L. (2012). Three-dimensional porous graphene-based composite materials: Electrochemical synthesis and application. J. Mater. Chem..

[25] Sheng K.X., Sun Y.Q., Li C., Yuan W.J., Shi G.Q. (2012). Ultrahigh-rate supercapacitors based on eletrochemically reduced graphene oxide for ac line-filtering. Sci. Rep..

[26] Cong H.P., Ren X.C., Wang P., Yu S.H. (2012). Macroscopic multifunctional graphene-based hydrogels and aerogels by a metal ion induced self-assembly process. ACS Nano.

[27] He Y.L., Li J.H., Li L.F., Chen J.B., Li J.Y. (2016). The synergy reduction and self-assembly of graphene oxide via gamma-ray irradiation in an ethanediamine aqueous solution. Nucl. Sci. Tech..

[28] Huang X.D., Sun B., Su D.W., Zhao D.Y., Wang G.X. (2014). Soft-template synthesis of 3D porous graphene foams with tunable architectures for lithium-O_2_ batteries and oil adsorption applications. J. Mater. Chem. A.

[29] Lee S.H., Kim H.W., Hwang J.O., Lee W.J., Kwon J., Bielawski C.W., Ruoff R.S., Kim S.O. (2010). Three-dimensional self-assembly of graphene oxide platelets into mechanically flexible macroporous carbon films. Angew. Chem. Int. Ed. Engl..

[30] Chen Z.P., Ren W.C., Gao L.B., Liu B.L., Pei S.F., Cheng H.M. (2011). Three-dimensional flexible and conductive interconnected graphene networks grown by chemical vapour deposition. Nat. Mater..

[31] Guo F., Jiang Y.Q., Xu Z., Xiao Y.H., Fang B., Liu Y.J., Gao W.W., Zhao P., Wang H.T., Gao C. (2018). Highly stretchable carbon aerogels. Nat. Commun..

[32] Gong Y., Yu Y.C., Kang H.X., Chen X.H., Liu H., Zhang Y., Sun Y.M., Song H.H. (2019). Synthesis and characterization of graphene oxide/chitosan composite aerogels with high mechanical performance. Polymers.

[33] Zhuo B., Cao S.A., Li X.P., Liang J.H., Bei Z.H., Yang Y.T., Yuan Q.P. (2020). A nanofibrillated cellulose-based electrothermal aerogel constructed with carbon nanotubes and graphene. Molecules.

[34] Weng W.S., Lin J., Du Y.C., Ge X.F., Zhou X.S., Bao J.C. (2018). Template-free synthesis of metal oxide hollow micro-/nanospheres via Ostwald ripening for lithium-ion batteries. J. Mater. Chem. A.

[35] Kim J., Cote L.J., Kim F., Yuan W., Shull K.R., Huang J. (2010). Graphene oxide sheets at interfaces. J. Am. Chem. Soc..

[36] Li C., Shi G.Q. (2012). Three-dimensional graphene architectures. Nanoscale.

[37] Luo J., Cote L.J., Tung V.C., Tan A.T., Goins P.E., Wu J., Huang J. (2010). Graphene oxide nanocolloids. J. Am. Chem. Soc..

[38] Boukhvalov D.W., Katsnelson M.I. (2008). Modeling of graphite oxide. J. Am. Chem. Soc..

[39] Erickson K., Erni R., Lee Z., Alem N., Gannett W., Zettl A. (2010). Determination of the local chemical structure of graphene oxide and reduced graphene oxide. Adv. Mater..

[40] Qin S.Y., Liu X.J., Zhuo R.X., Zhang X.Z. (2012). Microstructure-controllable graphene oxide hydrogel film based on a pH-responsive graphene oxide hydrogel. Macromol. Chem. Phys..

[41] Chen W.F., Yan L.F., Bangal P.R. (2010). Chemical reduction of graphene oxide to graphene by sulfur-containing compounds. J. Phys. Chem. C.

[42] Zhu X.J., Zhu Y.W., Murali S., Stoller M.D., Ruoff R.S. (2011). Nanostructured reduced graphene oxide/Fe_2_O_3_ composite as a high-performance anode material for lithium ion batteries. ACS Nano.

[43] Chen W.F., Li S.R., Chen C.H., Yan L.F. (2011). Self-assembly and embedding of nanoparticles by in situ reduced graphene for preparation of a 3D graphene/nanoparticle aerogel. Adv. Mater..

[44] Li J.H., Li J.Y., Meng H., Xie S.Y., Zhang B.W., Li L.F., Ma H.J., Zhang J.Y., Yu M. (2014). Ultra-light, compressible and fire-resistant graphene aerogel as a highly efficient and recyclable absorbent for organic liquids. J. Mater. Chem. A.

[45] Hu H., Zhao Z.B., Wan W.B., Gogotsi Y., Qiu J.S. (2013). Ultralight and highly compressible graphene aerogels. Adv. Mater..

[46] Sun H.Y., Xu Z., Gao C. (2013). Multifunctional, ultra-flyweight, synergistically assembled carbon aerogels. Adv. Mater..

[47] Acik M., Lee G., Mattevi C., Chhowalla M., Cho K., Chabal Y.J. (2010). Unusual infrared-absorption mechanism in thermally reduced graphene oxide. Nat. Mater..

[48] Akhavan O. (2010). The effect of heat treatment on formation of graphene thin films from graphene oxide nanosheets. Carbon.

[49] Kar T., Devivaraprasad R., Singh R.K., Bera B., Neergat M. (2014). Reduction of graphene oxide-a comprehensive electrochemical investigation in alkaline and acidic electrolytes. RSC Adv..

[50] Liang J.F., Liu Y.K., Guo L., Li L.D. (2013). Facile one-step synthesis of a 3D macroscopic SnO_2_-graphene aerogel and its application as a superior anode material for Li-ion batteries. RSC Adv..

[51] Niu Z.Q., Liu L.L., Zhang L., Shao Q., Zhou W.Y., Chen X.D., Xie S.S. (2014). A universal strategy to prepare functional porous graphene hybrid architectures. Adv. Mater..

[52] Whitby R.L. (2014). Chemical control of graphene architecture: Tailoring shape and properties. ACS Nano.

[53] Shi Q.R., Cha Y., Song Y., Lee J.I., Zhu C.Z., Li X.Y., Song M.K., Du D., Lin Y.H. (2016). 3D graphene-based hybrid materials: Synthesis and applications in energy storage and conversion. Nanoscale.

[54] Gao H.C., Sun Y.M., Zhou J.J., Xu R., Duan H.W. (2013). Mussel-inspired synthesis of polydopamine-functionalized graphene hydrogel as reusable adsorbents for water purification. ACS Appl. Mater. Inter..

[55] Tao Y., Kong D.B., Zhang C., Lv W., Wang M.X., Li B.H., Huang Z.H., Kang F.Y., Yang Q.H. (2014). Monolithic carbons with spheroidal and hierarchical pores produced by the linkage of functionalized graphene sheets. Carbon.

[56] Ye S.B., Feng J.C., Wu P.Y. (2013). Highly elastic graphene oxide-epoxy composite aerogels via simple freeze-drying and subsequent routine curing. J. Mater. Chem. A.

[57] Xu Y.X., Wu Q., Sun Y.Q., Bai H., Shi G.Q. (2010). Three-dimensional self-assembly of graphene oxide and DNA into multifunctional hydrogels. ACS Nano.

[58] Qin Y., Zhang Y., Qi N., Wang Q.Z., Zhang X.J., Li Y. (2019). Preparation of graphene aerogel with high mechanical stability and microwave absorption ability via combining surface support of metallic-CNTs and interfacial cross-linking by magnetic nanoparticles. ACS Appl. Mater. Inter..

[59] Arabkhani P., Asfaram A. (2020). Development of a novel three-dimensional magnetic polymer aerogel as an efficient adsorbent for malachite green removal. J. Hazard. Mater..

[60] Liu Y.D., Goebl J., Yin Y.D. (2013). Templated synthesis of nanostructured materials. Chem. Soc. Rev..

[61] Choi B.G., Yang M., Hong W.H., Choi J.W., Huh Y.S. (2012). 3D macroporous graphene frameworks for supercapacitors with high energy and power densities. ACS Nano.

[62] Wang X.J., Feng J., Bai Y.C., Zhang Q., Yin Y.D. (2016). Synthesis, properties, and applications of hollow micro-/nanostructures. Chem. Rev..

[63] Huang X.D., Qian K., Yang J., Zhang J., Li L., Yu C.Z., Zhao D.Y. (2012). Functional nanoporous graphene foams with controlled pore sizes. Adv. Mater..

[64] Meng Y., Gu D., Zhang F.Q., Shi Y.F., Yang H.F., Li Z., Yu C.Z., Tu B., Zhao D.Y. (2005). Ordered mesoporous polymers and homologous carbon frameworks: Amphiphilic surfactant templating and direct transformation. Angew. Chem. Int. Ed. Engl..

[65] Lu K.Q., Xin X., Zhang N., Tang Z.R., Xu Y.J. (2018). Photoredox catalysis over graphene aerogel-supported composites. J. Mater. Chem. A.

[66] Yin S.Y., Zhang Y.Y., Kong J.H., Zou C.J., Li C.M., Lu X.H., Ma J., Boey F.Y., Chen X.D. (2011). Assembly of graphene sheets into hierarchical structures for high-performance energy storage. ACS Nano.

[67] Hou X.F., Zheng Y.H., Ma X.L., Liu Y.H., Ma Z.C. (2021). The effects of hydrophobicity and textural properties on hexamethyldisiloxane adsorption in reduced graphene oxide aerogels. Molecules.

[68] Sui Z.Y., Meng Y.N., Xiao P.W., Zhao Z.Q., Wei Z.X., Han B.H. (2015). Nitrogen-doped graphene aerogels as efficient supercapacitor electrodes and gas adsorbents. ACS Appl. Mater. Inter..

[69] Hu H., Zhao Z.B., Gogotsi Y., Qiu J.S. (2014). Compressible carbon nanotube-graphene hybrid aerogels with superhydrophobicity and superoleophilicity for oil sorption. Environ. Sci. Tech. Let..

[70] Wang J., Fang F., Yuan T., Yang J.H., Chen L., Yao C., Zheng S.Y., Sun D.L. (2017). Three-dimensional graphene/single-walled carbon nanotube aerogel anchored with SnO_2_ nanoparticles for high performance lithium storage. ACS Appl. Mater. Inter..

[71] Wu Z.S., Yang S.B., Sun Y., Parvez K., Feng X.L., Mullen K. (2012). 3D nitrogen-doped graphene aerogel-supported Fe_3_O_4_ nanoparticles as efficient electrocatalysts for the oxygen reduction reaction. J. Am. Chem. Soc..

[72] Hu Y.J., Zhuo H., Chen Z.H., Wu K.Z., Luo Q.S., Liu Q.Z., Jing S.S., Liu C.F., Zhong L.X., Sun R.C. (2018). Superelastic carbon aerogel with ultrahigh and wide-range linear sensitivity. ACS Appl. Mater. Inter..

[73] Cao C.R., Yuan B.H. (2021). Thermally induced fire early warning aerogel with efficient thermal isolation and flame-retardant properties. Polym. Advan. Technol..

[74] Zuo B.Y., Yuan B.H. (2021). Flame-retardant cellulose nanofiber aerogel modified with graphene oxide and sodium montmorillonite and its fire-alarm application. Polym. Advan. Technol..

[75] Tong Z.W., Yang D., Shi J.F., Nan Y.H., Sun Y.Y., Jiang Z.Y. (2015). Three-dimensional porous aerogel constructed by g-C_3_N_4_ and graphene oxide nanosheets with excellent visible-light photocatalytic performance. ACS Appl. Mater. Inter..

[76] Ren L., Hui K.N., Hui K.S., Liu Y.D., Qi X., Zhong J.X., Du Y., Yang J.P. (2016). Corrigendum: 3D hierarchical porous graphene aerogel with tunable meso-pores on graphene nanosheets for high-performance energy storage. Sci. Rep..

[77] Chen Q.Y., Luo Z., Hills C., Xue G., Tyrer M. (2009). Precipitation of heavy metals from wastewater using simulated flue gas: Sequent additions of fly ash, lime and carbon dioxide. Water. Res..

[78] Chen Q.Y., Yao Y., Li X.Y., Lu J., Zhou J., Huang Z.L. (2018). Comparison of heavy metal removals from aqueous solutions by chemical precipitation and characteristics of precipitates. J. Water Process Eng..

[79] Tsai W.T., Chen H.R. (2010). Removal of malachite green from aqueous solution using low-cost chlorella-based biomass. J. Hazard. Mater..

[80] Doke S.M., Yadav G.D. (2014). Novelties of combustion synthesized titania ultrafiltration membrane in efficient removal of methylene blue dye from aqueous effluent. Chemosphere.

[81] Leo C.P., Chai W.K., Mohammad A.W., Qi Y., Hoedley A.F., Chai S.P. (2011). Phosphorus removal using nanofiltration membranes. Water. Sci. Technol..

[82] Akhurst D.J., Jones G.B., Clark M., McConchie D. (2006). Phosphate removal from aqueous solutions using neutralised bauxite refinery residues (Bauxsol™). Environ. Chem..

[83] Terry T.A. (2009). Removal of nitrates and phosphates by ion exchange with hydrotalcite. Environ. Eng. Sci..

[84] Nguyen D.D., Tai N.H., Lee S.B., Kuo W.S. (2012). Superhydrophobic and superoleophilic properties of graphene-based sponges fabricated using a facile dip coating method. Energy Environ. Sci..

[85] Tran D.N.H., Kabiri S., Wang L., Losic D. (2015). Engineered graphene-nanoparticle aerogel composites for efficient removal of phosphate from water. J. Mater. Chem. A.

[86] Gomez-Navarro C., Weitz R.T., Bittner A.M., Scolari M., Mews A., Burghard M., Kern K. (2007). Electronic transport properties of individual chemically reduced graphene oxide sheets. Nano. Lett..

[87] Wang H.L., Cui L.F., Yang Y., Sanchez Casalongue H., Robinson J.T., Liang Y.Y., Cui Y., Dai H.J. (2010). Mn_3_O_4_-graphene hybrid as a high-capacity anode material for lithium ion batteries. J. Am. Chem. Soc..

[88] Zhou G.M., Wang D.W., Li F., Zhang L.L., Li N., Wu Z.S., Wen L., Lu G.Q., Cheng H.M. (2010). Graphene-wrapped Fe_3_O_4_ anode material with improved reversible capacity and cyclic stability for lithium ion batteries. Chem. Mater..

[89] Ji L.W., Tan Z.K., Kuykendall T.R., Aloni S., Xun S.D., Lin E., Battaglia V., Zhang Y.G. (2011). Fe_3_O_4_ nanoparticle-integrated graphene sheets for high-performance half and full lithium ion cells. Phys. Chem. Chem. Phys..

[90] Askari M.B., Salarizadeh P., Seifi M., Ramezan zadeh M.H., Di Bartolomeo A. (2021). ZnFe_2_O_4_ nanorods on reduced graphene oxide as advanced supercapacitor electrodes. J. Alloys Compd..

[91] Zhang M., Lei D.N., Yin X.M., Chen L.B., Li Q.H., Wang Y.G., Wang T.H. (2010). Magnetite/graphene composites: Microwave irradiation synthesis and enhanced cycling and rate performances for lithium ion batteries. J. Mater. Chem..

[92] Dikin D.A., Stankovich S., Zimney E.J., Piner R.D., Dommett G.H., Evmenenko G., Nguyen S.T., Ruoff R.S. (2007). Preparation and characterization of graphene oxide paper. Nature.

[93] Kim K.H., Oh Y., Islam M.F. (2012). Graphene coating makes carbon nanotube aerogels superelastic and resistant to fatigue. Nat. Nano-Technol..

[94] Qiu L., Liu J.Z., Chang S.L., Wu Y.Z., Li D. (2012). Biomimetic superelastic graphene-based cellular monoliths. Nat. Commun..

[95] Yilmaz G., Lu X.M., Ho G.W. (2017). Cross-linker mediated formation of sulfur-functionalized V_2_O_5_/graphene aerogels and their enhanced pseudocapacitive performance. Nanoscale.

[96] Lee B., Lee S., Lee M., Jeong D.H., Baek Y., Yoon J., Kim Y.H. (2015). Carbon nanotube-bonded graphene hybrid aerogels and their application to water purification. Nanoscale.

[97] Tang Y., Gong S., Chen Y., Yap L.W., Cheng W.L. (2014). Manufacturable conducting rubber ambers and stretchable conductors from copper nanowire aerogel monoliths. ACS Nano.

[98] Si Y., Yu J.Y., Tang X.M., Ge J.L., Ding B. (2014). Ultralight nanofibre-assembled cellular aerogels with superelasticity and multifunctionality. Nat. Commun..

[99] Qi H.S., Mäder E., Liu J.W. (2013). Electrically conductive aerogels composed of cellulose and carbon nanotubes. J. Mater. Chem. A.

[100] Fratzl P., Weinkamer R. (2007). Nature’s hierarchical materials. Prog. Mater. Sci..

[101] Xie H.L., Lai X.J., Li H.Q., Gao J.F., Zeng X.R., Huang X.Y., Lin X.Y. (2019). A highly efficient flame retardant nacre-inspired nanocoating with ultrasensitive fire-warning and self-healing capabilities. Chem. Eng. J..

[102] Evans D.D., Stroup D.W. (1986). Methods to calculate the response time of heat and smoke detectors installed below large unobstructed ceilings. Fire Technol..

[103] Qualey Iii J.R. (2000). Fire test comparisons of smoke detector response times. Fire Technol..

[104] Xie H.L., Lai X.J., Li H.Q., Gao J.F., Zeng X.R., Huang X.Y., Zhang S.F. (2020). A sandwich-like flame retardant nanocoating for supersensitive fire-warning. Chem. Eng. J..

[105] Chen J.Y., Xie H.L., Lai X.J., Li H.Q., Gao J.F., Zeng X.R. (2020). An ultrasensitive fire-warning chitosan/montmorillonite/carbon nanotube composite aerogel with high fire-resistance. Chem. Eng. J..

[106] Chen Z.H., Hu Y.J., Zhuo H., Liu L.X., Jing S.S., Zhong L.X., Peng X.W., Sun R.C. (2019). Compressible, elastic, and pressure-sensitive carbon aerogels derived from 2D titanium carbide nanosheets and bacterial cellulose for wearable sensors. Chem. Mater..

[107] Pang Y., Zhang K.N., Yang Z., Jiang S., Ju Z.Y., Li Y.X., Wang X.F., Wang D.Y., Jian M.Q., Zhang Y.Y. (2018). Epidermis microstructure inspired graphene pressure sensor with random distributed spinosum for high sensitivity and large linearity. ACS Nano.

[108] Zhang N., Yang M.Q., Liu S.Q., Sun Y.G., Xu Y.J. (2015). Waltzing with the versatile platform of graphene to synthesize composite photocatalysts. Chem. Rev..

[109] Wan W.C., Yu S., Dong F., Zhang Q., Zhou Y. (2016). Efficient C_3_N_4_/graphene oxide macroscopic aerogel visible-light photocatalyst. J. Mater. Chem. A.

[110] Park J., Jin T., Liu C., Li G.H., Yan M.D. (2016). Three-dimensional graphene-TiO_2_ nanocomposite photocatalyst synthesized by covalent attachment. ACS Omega.

[111] Yang M.Q., Zhang N., Wang Y., Xu Y.J. (2017). Metal-free, robust, and regenerable 3D graphene-organics aerogel with high and stable photosensitization efficiency. J. Catal..

[112] Gao M.M., Peh C.K.N., Ong W.L., Ho G.W. (2013). Green chemistry synthesis of a nanocomposite graphene hydrogel with three-dimensional nano-mesopores for photocatalytic H_2_ production. RSC Adv..

[113] Wan W.C., Lin Y.H., Prakash A., Zhou Y. (2016). Three-dimensional carbon-based architectures for oil remediation: From synthesis and modification to functionalization. J. Mater. Chem. A.

[114] Fan Y.Y., Ma W.G., Han D.X., Gan S.Y., Dong X.D., Niu L. (2015). Convenient recycling of 3D AgX/graphene aerogels (X = Br, Cl) for efficient photocatalytic degradation of water pollutants. Adv. Mater..

[115] Zhou H., Li P., Guo J.J., Yan R.Y., Fan T.X., Zhang D., Ye J.H. (2015). Artificial photosynthesis on tree trunk derived alkaline tantalates with hierarchical anatomy: Towards CO_2_ photo-fixation into CO and CH_4_. Nanoscale.

[116] Garcia-Gonzalez C.A., Sosnik A., Kalmar J., De Marco I., Erkey C., Concheiro A., Alvarez-Lorenzo C. (2021). Aerogels in drug delivery: From design to application. J. Control. Release.

[117] Zaman A., Huang F., Jiang M., Wei W., Zhou Z.W. (2020). Preparation, properties, and applications of natural cellulosic aerogels: A review. Energy. Built. Environ..

